# Smart pH-sensitive nanoassemblies with cleavable PEGylation for tumor targeted drug delivery

**DOI:** 10.1038/s41598-017-03111-2

**Published:** 2017-06-13

**Authors:** Guanren Zhao, Ling Long, Lina Zhang, Mingli Peng, Ting Cui, Xiaoxun Wen, Xing Zhou, Lijun Sun, Ling Che

**Affiliations:** 1 Department of Pharmacy, Hospital 309 of PLA, Beijing, 100091 China; 2 Department of oncology, Xinqiao Hospital, Third Military Medical University, Chongqing, 400038 China; 3 Department of Pharmaceutics, College of Pharmacy, Third Military Medical University, Chongqing, 400038 China

## Abstract

A new acidly sensitive PEGylated polyethylenimine linked by Schiff base (PEG-s-PEI) was designed to render pH-sensitive PEGylation nanoassemblies through multiple interactions with indomethacin and docetaxel (DTX). DTX nanoassemblies driven by PEG-s-PEI thus formulated exhibited an excellent pH-sensitivity PEGylation cleavage performance at extracellular pH of tumor microenvironment, compared to normal tissues, thereby long circulated in blood but were highly phagocytosed by tumor cells. Consequently, this smart pH-sensitive PEGylation cleavage provided an efficient strategy to target tumor microenvironment, in turn afforded superior therapeutic outcome in anti-tumor activity.

## Introduction

Polyethylene glycol (PEG) is widely used to coat the surface of the therapeutic as “stealth” polymer for increasing circulation time of drugs^1–9^. PEGylation of nanoparticles (NPs) has been investigated to prevent nanoparticles from clearance by the cells of the mononuclear phagocyte system (MPS) since the early 80s^10–14^, which can prevent NPs from aggregation, opsonization, and phagocytosis, thereby prolonging circulation time and increasing NPs targeted to tumor sites by enhanced permeability and retention (EPR) effect^10, 15, 16^. Meanwhile, PEGylation also reduces the desired binding to the negatively charged cell membrane for entering into tumor cells, which is preferred positive surface charges^17, 18^. However, systemic application of positively charged NPs would result in significant toxicity and/or poor efficiency due to binding to plasma proteins or blood cells and complement activation with significant clearance by MPS^19^. Obviously, sensitively cleavable PEGylation of positively charged NPs, with PEG chains stretching in circulation and resident tissues, but cleaved in tumor microenvironment, may exhibit long blood circulation time with efficient phagocytosis by tumor cells^20–24^.

Contributed by high rate of glycolysis in cancer cells, the extracellular pH (pH_e_) of tumor microenvironment shows an acidy pH value mainly at 6.9–7.2 and lowest at 5.7. In contrast, the pH_e_ of normal tissues is constantly kept at pH 7.4^25–27^. Based on pH_e_ gradient between normal tissues and tumor microenvironment, as well as the interior of endosomes (usually more acidic), pH-sensitive polymers and pH-sensitive NPs have been designed to facilitate the release of anticancer drugs in a pH-controlled manner^28–34^. Meanwhile, pH sensitive cleavage of PEGylation was also succeed applied in gene delivery and liposome coating for long blood circulation time and efficient phagocytosis by tumor cells^17, 18, 35^. However, in line with the challenges facing the new delivery system, there are still enormous challenges in successful bench to bedside translation of the plethora of PEGylation cleavable nanotherapeutics developed in laboratory, limited by their low drug loading capacity, complicated materials synthesis, and hardly reproducible manufacturing. Furthermore, the high cost is another factor, which limits the successful translation of these delivery systems^36, 37^. Consequently, there is still unmet demand for developing facile, cost-effective and powerful approaches with good scalability and consistency in terms of manufacturing to produce PEGylation cleavable nanotherapeutics with versatile functions and broad applications.

In our previous studies, we discovered a one-pot and high efficient fabrication of polymer nanotherapeutics based on commercially available homopolymers (such as polyethyleneimine (PEI)) and small molecule drugs through multiple interactions mediated self-assembly, with high drug loading capacity and desirable therapeutic benefits, which showed great potential and advantages in clinical transformation as efficient oral nanocontainers for other hydrophobic drugs^38, 39^. To combine this system with PEGylation cleavable profile is expected to integrate its advantages to help clinical transformation of PEGylation cleavable nanotherapeutics. Therefore, we developed a new acidly sensitive PEGylation cleavable PEI linked by Schiff base which is used to render pH-sensitive PEGylated NAs through multiple interactions with small molecule drugs mediated self-assembly in this study. Nanoparticles thus produced, with facile material synthesis, high drug loading capacity, desirable therapeutic benefits, low toxicity for intravenous application and pH-triggered deshielding of PEG, can serve as efficient and tumor environment targeting nanocontainers for anti-cancer drugs, and conducive to clinical transformation of PEGylation cleavable nanotherapeutics.

## Results

### Synthesis and characterization of polymers

**Figure 1:**
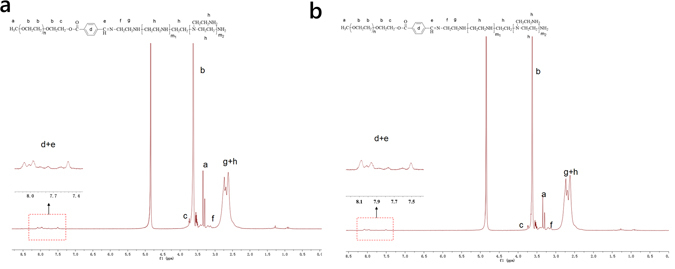
^1^H-NMR spectrum spectras of PEG-s-PEI-1 (**a**) and PEG-s-PEI-2 (**b**) in CH_2_DOH.

The synthesis of PEGylated PEIs linked by Schiff base (PEG-s-PEIs) were carried out at various PEG/PEI monomeric molar ratios for PEG-s-PEI-1 (2/1), PEG-s-PEI-2 (4/1) and PEG-s-PEI-3 (8/1). PEGylated PEI linked by amide linkage (PEG-b-PEI) was synthesized at PEG/PEI monomeric molar ratio of 4/1. Disappointingly, PEG-s-PEI-3 synthesized was cross-linked to be not soluble in water or any organic solvent we tried. The synthesis of mPEG-CHO (Fig. S1b), PEG-b-PEI (Fig. S1c), PEG-s-PEI-1 (Fig. 1a) and PEG-s-PEI-2 (Fig. 1b) were determined by ^1^H NMR. The number of PEG linked at PEI was calculated that one PEG-CHO segment was linked at every 22, 18 and 16 PEI monomeric units for PEG-s-PEI-1, PEG-s-PEI-2 and PEG-b-PEI respectively. Molecular weight information of various polymers mentioned in this study was obtained by GPC measurement (Table S1 and Fig. S1d–f). The amounts of unreacted PEG remained in PEG-s-PEIs were determined as 1.35% and 1.12% for PEG-s-PEI-1 and PEG-s-PEI-2, respective.

Hydrolysis of PEG-s-PEIs were carried out that half-life times of PEG-s-PEIs were more than 5 h at pH 7.4, but less than 25 min at lower pH. The half-life times of PEG-s-PEI-1 and PEG-s-PEI-2 were 90 min and 2 h at pH 6.5, respectively (Fig. S2a,b). And the cytotoxicity of PEG-s-PEI-2 against HepG2 was significantly decreased than PEI, either in pH 6.5 or pH 7.4 mediums (Fig. S2c,d).

### Computational design and assembly of DTX/IND/PEG-s-PEI

**Figure 2:**
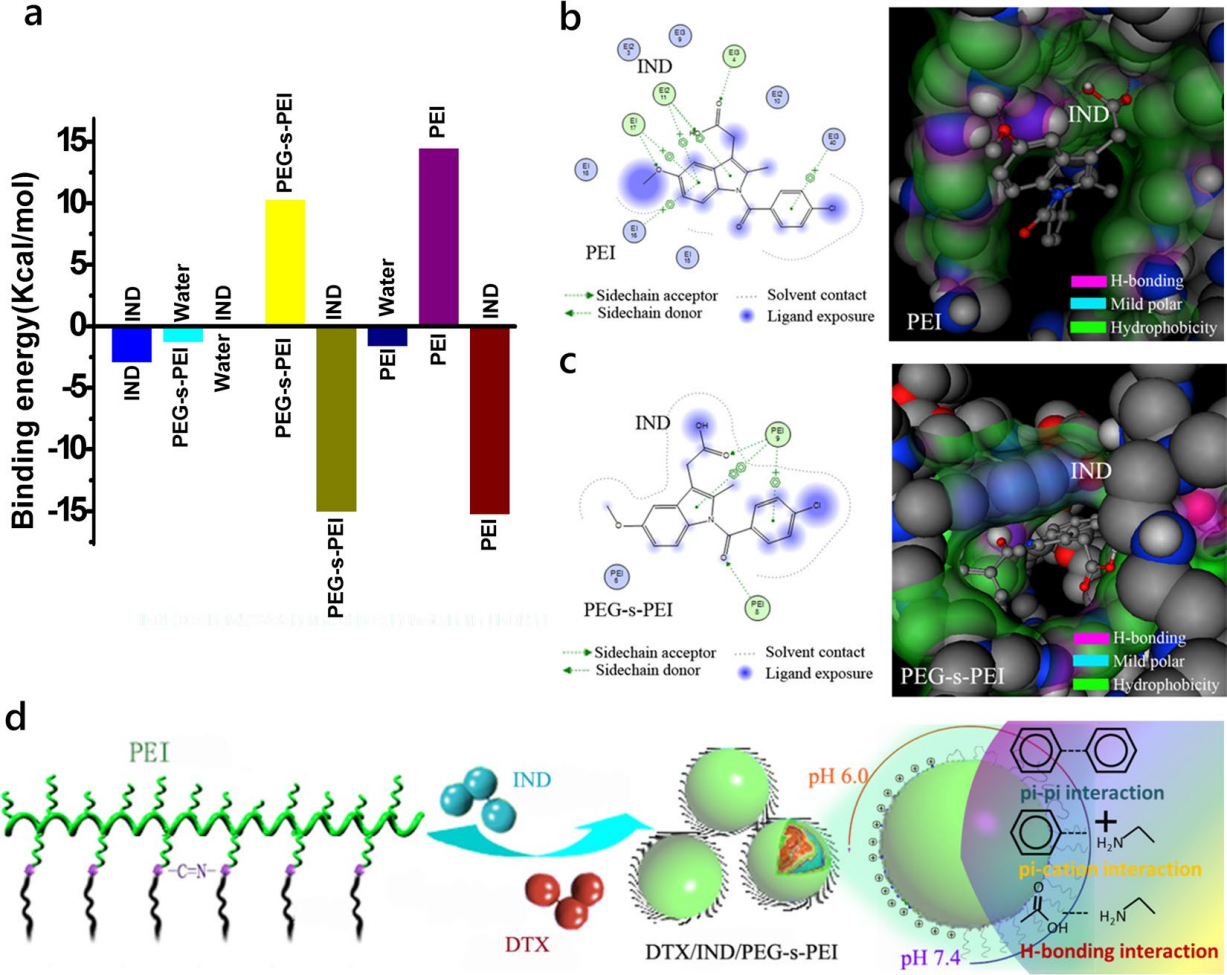
PEI and PEG-s-PEI interactions with IND based on the docking into ploymers. (**a**) The calculated bind energy of various components in IND/polymer mixtures stimulated by computational simulation. (**b**,**c**) Lowest energy 2D (left) and 3D (right) comformation of IND in case of IND docked into PEI (**b**) and PEG-s-PEI (**c**). (**d**) Schematic drawing of pH-sensitive PEGlyation cleavable PEGlyated nanoassemblies assembled by DTX, IND and PEG-s-PEI. In 3d styles, the color code for the drug and polymer is based on the atom type: in gray are the carbons, in red oxygen atoms, in dark blue are nitrogen atoms and in light gray are the polar hydrogen atoms that are forming H-bonds. The surface sites of interaction was drawn and colored by Connolly method built in MOE.

Initially, indomethacin (IND), a widely used nonsteroidal anti-inflammatory drug (NSAID), was utilized as guest molecular to dock into PEG-s-PEI or PEI. IND and PEG-s-PEI displayed a little lower intermolecular energy compared to IND and PEI interaction. However, it is significantly higher than that of other combinations in IND/PEG-s-PEI system (Fig. 2a). The lowest energy conformations of IND within PEG-s-PEI chains or PEI chains after the docking calculations in 3D conformation were both exactly “embraced” within big pockets with H-bonding and hydrophobic areas formed by the sidechains and backbone atoms of PEG-s-PEI or PEI (Fig. 2b,c).

**Figure 3:**
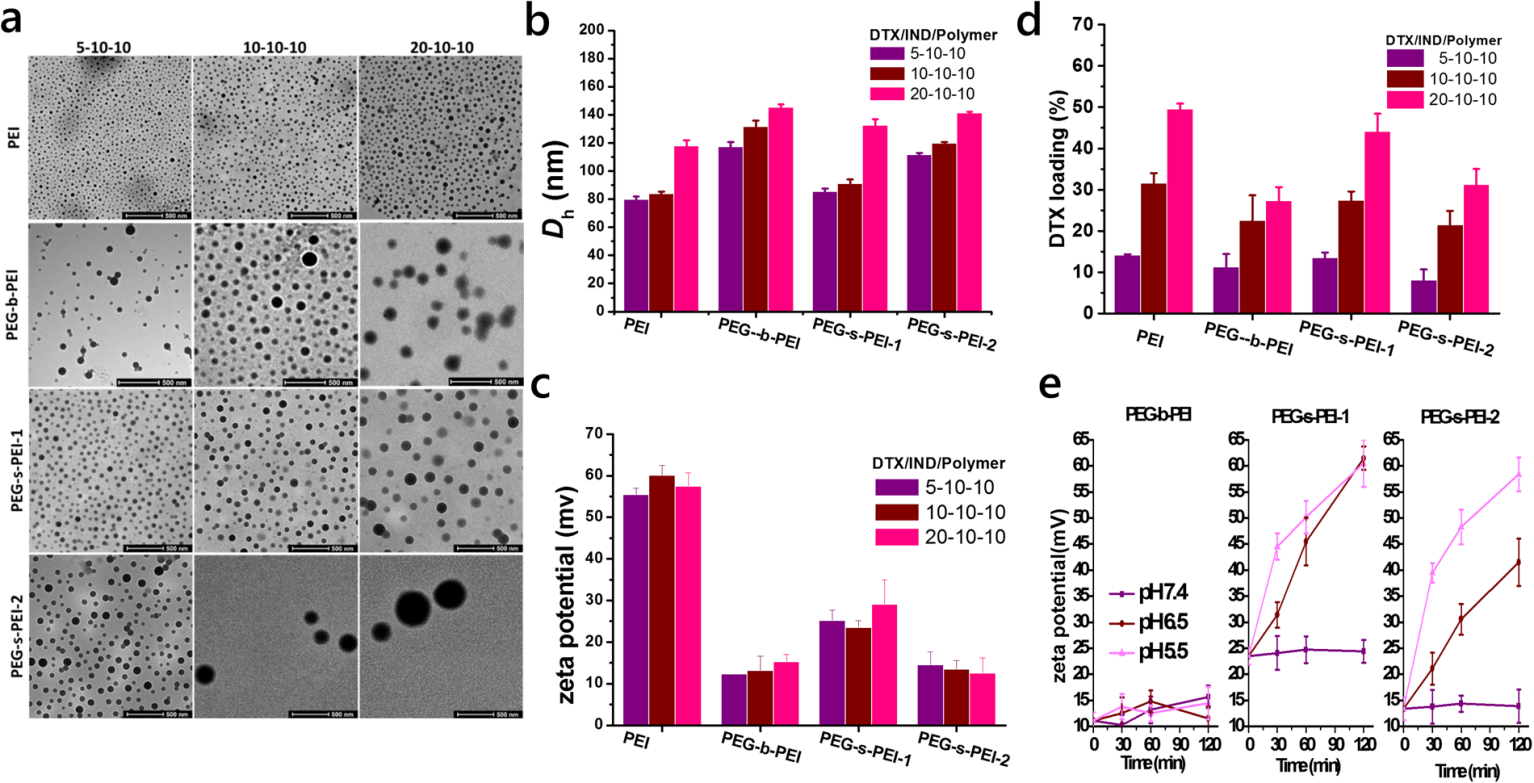
Characterization of physicochemical properties of NAs formed by DTX,IND and Polymers at various weight ratios. (**a**) TME images. (**b**) Hydrodynamic diameter (*D*_h_). (**c**) ζ-potential. (**d**) DTX loading contents in NAs. (**e**) Changes in ζ-potential in various PBS buffers at pH 7.4, 6.5 and 5.5.

Based on computational results, self-assemblies of DTX, IND and PEG-s-PEI with various DTX/IND/PEG-s-PEIs weight ratios were conducted by dialysis against deionized water using methanol as the common solvent^40^. Independent of DTX/IND/Polymer weight ratio, spherical nanoassemblies were assembled with monodispersed distribution profiles (Fig. 3a). The hydrodynamic diameter (*D*_h_) of assemblies increased when the PEG/PEI molar ratio increased (Fig. 3b), while ζ-potential decreased (Fig. 3c). HPLC quantification revealed a dramatic enhancement in DTX loading content when drug feeding ratios of DTX/IND/PEG-s-PEIs increased (Fig. 3d), while IND loading contents were correspondingly reduced (Fig. S3). Compared with DTX/IND/PEI NAs, the hemolysis ratio of DTX/IND-PEG-s-PEI-1, DTX/IND/PEG-s-PEI-2 and DTX/IND/PEG-b-PEI was lowered to 11.34 ± 1.56, 3.231 ± 0.822 and 1.561 ± 0.351, respectively (Table S2).

### pH triggered PEGylation cleavage with potential shift

DTX/IND/PEG-s-PEI NAs at weight ratio of 10:10:10 were employed to study pH triggered PEGylation cleavage (Fig. 3e). At pH 5.5 and 6.5, DTX/IND/PEG-s-PEI NAs formed significantly surface potential shifting in 120 min, while stably retained low surface charge for the whole 2 h time period at pH 7.4. On the contrary, stable surface charge of DTX/IND/PEI NAs was maintained at 60 mV in all buffers even after 24 h later.

### Intracellular uptake of DTX Nanoassemblies and *in vitro* anti-tumor activity

**Figure 4:**
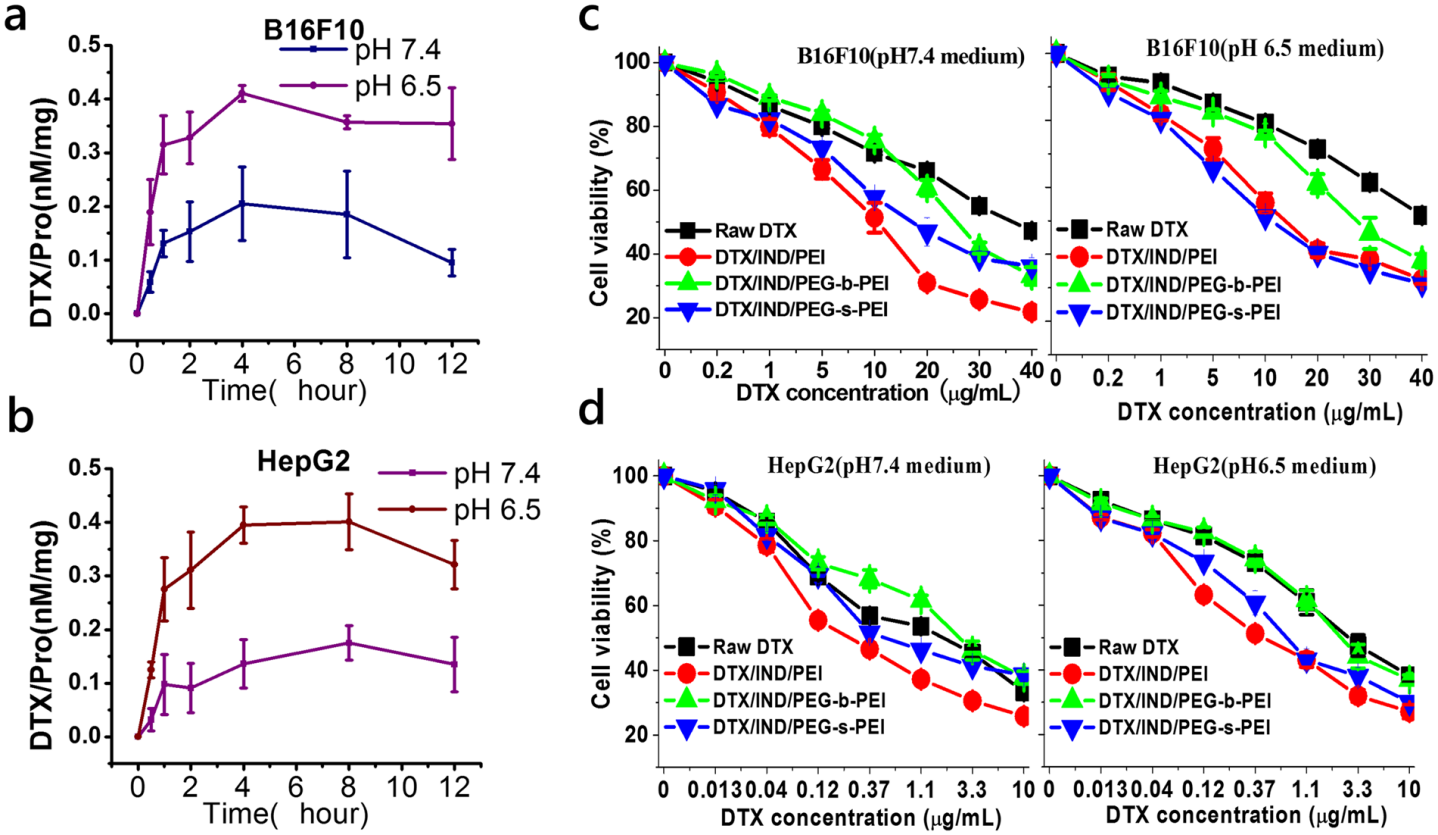
Cellular retention of internalized drug in tumor cells after endocytosis. (**a**,**b**) Time dependent DTX concentration changes after DTX/IND/PEG-s-PEI endocyozied by B16F10 (**a**) and HepG2 (**b**) in normal and acidy mediums. (**c**,**d**) *In vitro* antitumor activity of DTX nanomedicines formulated from DTX/IND/PEG-s-PEI assemblies against B16F10 (**c**) and HepG2 (**d**) in normal and acidy mediums. Data are mean ± S.E. (standard error, n = 6).

DTX/IND/PEG-s-PEI-2 NAs could be internalized by tumor cells such as B16F10 murine melanoma cancer cells (Fig. 4a) and HepG2 hepatocellular carcinoma cells (Fig. 4b) at high efficiency in acidy medium (pH 6.5), but lower efficiency in normal medium (pH 7.4). In contrast, the uptake of raw DTX, DTX/IND/PEG-b-PEI and DTX/IND/PEI NAs by tumor cells were equal either at pH 7.4 or pH 6.5 (Fig. S4), that DTX/IND/PEI NAs were highly internalized in B16F10 and HepG2 cells, raw DTX and DTX/IND/PEG-b-PEI NAs were little internalized in cells, comparably (Fig. S4).

Evaluation on *in vitro* antitumor activities by MTT assay showed the superior efficacy of assembled DTX/IND/PEI and DTX/IND/PEG-s-PEI NAs over raw DTX against various cancer cells including B16F10 and HepG2 in acidy and normal mediums. However, the cytotoxicity of DTX/IND/PEG-s-PEI NAs, that is lower than DTX/IND/PEI NAs in normal medium, was shifted to be no difference with DTX/IND/PEI NAs in acidy medium (Figs 4c,d and S5a,b). Interestingly, the cytotoxicity of raw DTX, DTX/IND/PEG-b-PEI and DTX/IND/PEI were significantly decreased in acidy medium, but the cytotoxicity of DTX/IND/PEG-s-PEI NAs was not effected^41^. The IC_50_ of DTX/IND/PEG-s-PEI NAs for tumor cells incubated in pH 6.5 medium was significantly lower than those incubated in pH 7.4 medium (Fig. S5e). DTX/IND/PEI NAs performed lower IC_50_ than other DTX formations when cells incubated in pH 7.4 medium (Fig. S5c), but similar anti-tumor activity as DTX/IND/PEI NAs was induced when DTX/IND/PEG-s-PEI NAs against tumor cells in pH 6.5 medium (Fig. S5d,e).

### *In Vitro* Release and *In Vivo* Pharmacokinetic Studies

**Figure 5:**
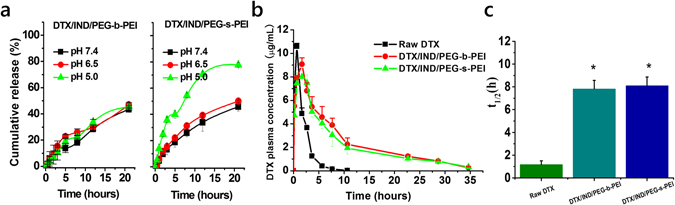
*In vitro* release and *in vivo* pharmacokinetic profiles of DTX/IND/PEG-s-PEI nanomedicines. (**a**) *In vitro* release profiles of DTX nanomedicines in the buffer simulating various pH conditions. Data are mean ± S.D. (n = 3). (**b**,**c**) Plasma concentrations (**b**) and t_1/2_ of DTX of various DTX-loaded nanomedicines (**c**) after injected in SD rats at 10 mg/kg of DTX. Data are mean ± S.D. (n = 5). *p < 0.05 versus﻿ Raw DTX.

Under *in vitro* conditions, DTX/IND/PEG-s-PEI NAs showed considerably rapid release at pH 5.0, slower release at pH 6.5, and further slower at pH 7.4 (Fig. 5a). Comparatively, no difference was found when DTX/IND/PEG-b-PEI NAs were employed to be released *in vitro* conditions (Fig. 5a). Subsequently, *in vivo* pharmacokinetic study carried out (Fig. 5b), the t_1/2_ of DTX/IND/PEG-s-PEI NAs and DTX/IND/PEG-b-PEI NAs were remarkably enhanced (Fig. 5c).

### Targeting delivery DTX to tumor for cancer therapy

**Figure 6:**
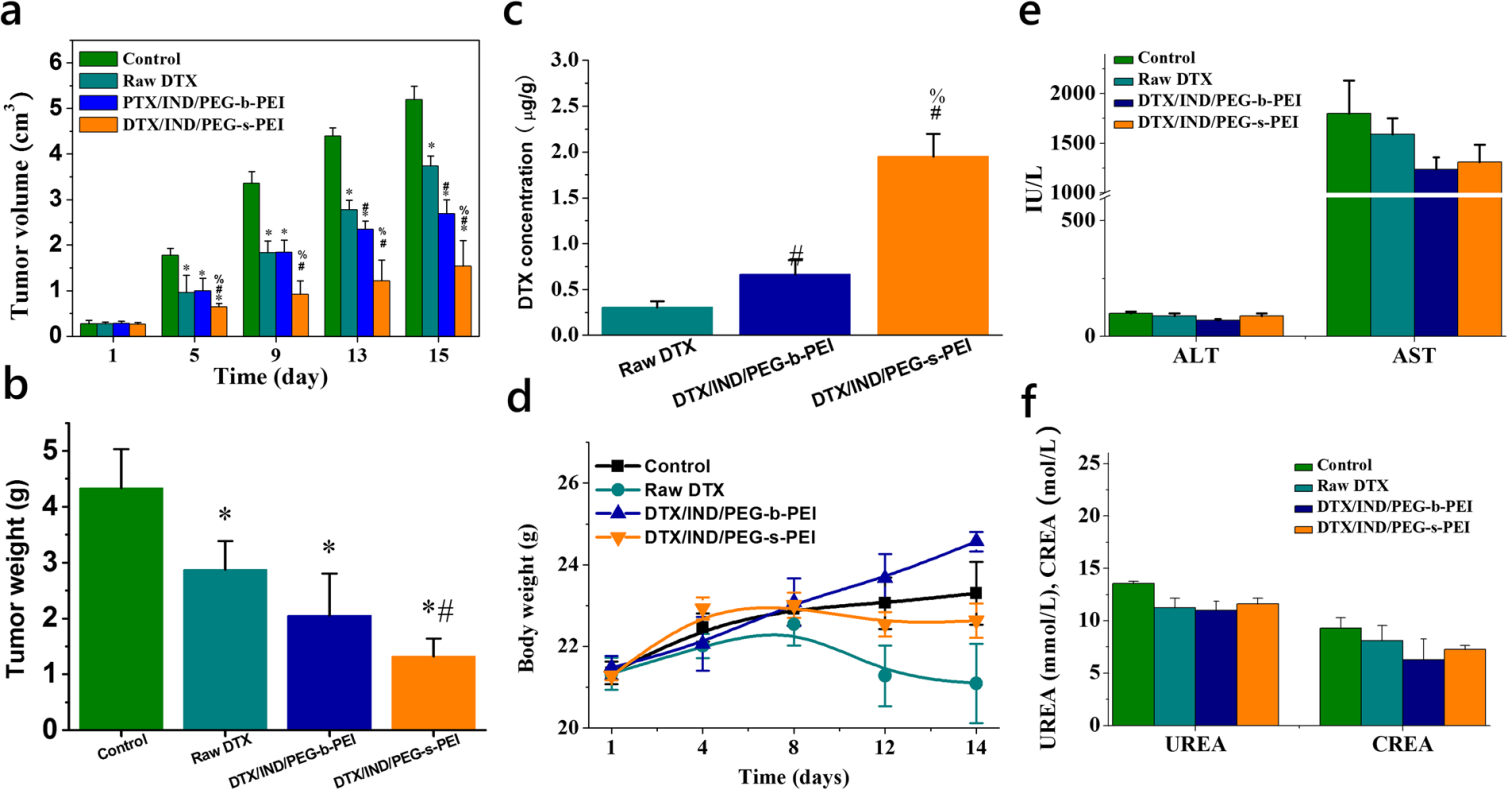
Improved therapeutic performance of DTX loaded nanomedicines. (**a**) Changes in the tumor volume of mice during the treatment. (**b**,**c**) The weight (**b**) and DTX concentration (**c**) of tumors excised from mice bearing melanoma xenografts 14 days after treatment with different DTX microspheres by oral gavage at 10 mg/kg of DTX every four days. (**d**) Changes in the body weight of mice during the treatment. (**e**,**f**) Typical biochemical markers relevant to liver (**e**) and kidney (**f**) functions. In all images, data are mean ± S.D. (n = 6). *p < 0.05 versus the model control; ^#^p < 0.05 versus Raw DTX; and ^%^p < 0.05 versus DTX/IND/PEG-b-PEI.

During the whole treatment, we monitored no abnormal change in the body weight of animals treated (Fig. 6d). However, significant differences on tumor volumes were observed for different groups (Fig. 6a). Treatment with DTX/IND/PEG-s-PEI NAs dramatically inhibited the tumor volume. Compared with raw DTX and DTX/IND/PEG-b-PEI NAs, DTX/IND/PEG-s-PEI NAs showed significant at the 5th, 9th, 13th and 15th days of treatment (Fig. 6a). And tumor weight of mice treated with DTX/IND/PEG-s-PEI NAs therapeutics based on PEG-s-PEI was significantly lower than that of mice administered with corresponding DTX/IND/PEG-b-PEI NAs and raw DTX (Fig. 6b). DTX concentration in tumors of DTX/IND/PEG-s-PEI treated group was much higher than that of other groups treated with raw DTX and DTX/IND/PEG-b-PEI (Fig. 6c).

The plasma levels of typical biomarkers including alanine aminotransferase (ALT), aspartate aminotransferase (AST), blood urea (UREA) and creatinine (CREA), which are relevant to liver and kidney functions, showed no significant increase (Fig. 6e,f). Additionally, we did not detect evident injuries or abnormalities in H&E sections of major organs such as heart, liver, spleen, lung and kidney (Fig. S6a), and no significant difference was found in organ index and Hematological parameters (Fig. S6b,c).

## Discussion

Cleavable PEGylation is a hot topic in drug delivery system to give vehicle long blood circulation time and efficient phagocytosis by tumor cells. However, low drug loading capacity, complicated materials synthesis, high cost and hardly reproducible manufacturing are limiting its successful translation from bench to bedside, which is also an enormously challenge in the whole novel drug delivery system. In our previous studies, we constructed a facile, convenient, cost-effective and easily scalable one-pot strategy to assemble various lipophilic therapeutics into nanomedicines, through which highly effective cargo loading and nanoparticles formation can be reached simultaneously. Besides dramatically improving the water solubility, the assembled nanopharmaceuticals show significantly higher bioavailability and better therapeutic activity. This strategy brings the dawn to translate novel drug delivery systems in laboratory from bench to bedside. Therefore, we hypothesize that PEGylation cleavable nanotherapeutics with high drug loading capacity, long blood circulation time and efficient phagocytosis by tumor cells, can be easily constructed by combining cleavable PEGylation with this facile, convenient, cost-effective and easily scalable one-pot self-assembly strategy discovered by our previous studies. In order to validate our hypothesis, we constructed a tumor targeting system of docetaxel based on multiple interactions between indomethacin and PEGylation cleavable PEI (PEG-s-PEI). In this case, IND/PEG-s-PEI nanoassemblies functioned as nanovehicles for DTX, and IND was also employed as COX inhibitor to reverse anorexia induced by docetaxel-based chemotherapy^42^, leading to an IND-DTX combined nanotherapy.

In this conceptual proof study, PEGylation cleavable PEI was firstly synthesized based on pH-sensitive linkage Schiff base and PEI, a commercially available homopolymer that has been widely utilized for both *in vitro* and *in vivo* gene delivery^43–45^, and also been used as a potent mucosal adjuvant for viral glycoprotein antigens most recently^46^. To determine a most suitable PEG-s-PEIs used in our study, three PEG-s-PEIs with PEG/PEI monomeric molar ratios 2/1 (PEG-s-PEI -1), 4/1 (PEG-s-PEI-2) and 8/1 (PEG-s-PEI-3) were synthesized. PEG-s-PEI -3 was cross-linked to be insoluble, which was firstly abandoned. PEG-s-PEI -1 and PEG-s-PEI-2 were successfully synthesized with well soluble ability in menthol and water, and both showed rapid pH sensitive cleavage of PEGylation from PEG-s-PEIs in acidly environment containing pH 5.5 to pH 6.5 (Fig. S2).

Guided by PEI may interact with small molecules containing carboxyl group to render NAs by multiple noncovalent forces to serve as efficient nanocontainers for other hydrophobic drugs, we found that there were similar interactions between IND and PEG-s-PEI by Autodock programs. Though PEGlyation decreased the electrostatic forces between PEG-s-PEI and IND, pi-pi stacks were supplemented by benzene rings in PEG-s-PEI. So PEG-s-PEI only displayed slightly lower intermolecular energy compared to IND and PEI interaction (Fig. 2c,d), that was notably higher than that of other combinations in IND/PEG-s-PEI system (Fig. 2a). Consistent with the computational results, DTX/IND/PEG-s-PEI-1 and DTX/IND/PEG-s-PEI-2 NAs at various DTX/IND/PEG-s-PEIs weight ratios were successfully conducted by dialysis against deionized water using methanol as the common solvent that spherical nanostructures with narrowly dispersed size were observed by TEM (Fig. 3a). Conducive to reproducible manufacturing and quality control, the size, ζ-potential and drug loading contents of these NAs could be easily controlled by DTX feeding (Fig. 3b–d). Contributed by PEGylation cleavage of PEG-s-PEIs in acidic conditions (Fig. S2), we monitored the pH-sensitive zeta potential shifting of DTX/IND/PEG-s-PEIs NAs at pH 5.5 and pH 6.5 (Fig. 4a,b), which imply that exposure of DTX/IND/PEG-s-PEIs NAs to pH 5.5 and pH 6.5 triggered the cleavage of PEGylation, and further led to strong shift of zeta potential of NAs. Importantly, NPs derived from PEI cannot be used in intravenous injection owned to the lethal toxicity of PEI leaded by hemolysis and thrombus rapidly formatted, which greatly limit the clinical transformation of these NPs. The PEGlyation of PEI was proved to improve the biocompatibility of PEI, such as hemolysis ratio (Table S2) and cytotoxicity (Fig. S2c,d). Despite the hemolysis ratio of DTX/IND/PEG-s-PEI-1 was lower than that of DTX/IND/PEI, it was still in dangerous degree, DTX/IND/PEG-s-PEI-2 showed a safe hemolysis ratio to contact with blood that was contributed by the higher PEG/PEI monomeric molar ratio (Table S2), which was employed in followed *in vitro* and *in vivo* experiments.

We next verified that the deshielding of PEGylation at pH 6.5 would facilitate the cellular internalization of NAs and increase DTX accumulation in tumor cells, leading to superior anti-tumor activity (Figs 4 and S4,5). DTX/IND/PEG-s-PEI-2 NAs showed significantly lower anti-tumor activity than DTX/IND/PEG-b-PEI NAs in normal medium, but comparable anti-tumor activity to DTX/IND/PEG-b-PEI NAs in acidy medium. As we know, acidic extracellular microenvironment was proved to decrease the chemo-sensitivity of tumor cells^47, 48^. We found the cytotoxicity of raw DTX, DTX/IND/PEG-b-PEI and DTX/IND/PEI were significantly decreased in acidy medium. In this case, the anti-tumor activities of DTX/IND/PEG-s-PEI-2 NAs at low DTX concentrations in acidy medium was inhibited with no improvement than normal medium, even though the DTX accumulation in tumor cells was significantly increased. However, the anti-tumor activities of DTX/IND/PEG-s-PEI-2 NAs at high concentrations were higher in acidy medium than normal, and significant difference between IC_50_ at pH 6.5 and 7.4 was still observed for DTX/IND/PEG-s-PEI-2 NAs, which was contributed by the more DTX accumulated in tumor cells that offset the impact of acidic environment on chemo-sensitivity.

To interrogate the drug delivery capacity of DTX NAs, both *in vitro* and *in vivo* evaluations were performed. Computational results showed that IND was “embraced” by phenyl group and imine chains of PEG-s-PEI, when PEGlyation was cleaved from PEG-s-PEI-2 in acidy medium, the pi-pi stacking between phenyl group of PEG-s-PEI-2 and IND was abolished that one part of IND molecular would be exposed, and the interaction force between IND and PEG-s-PEI faced the risk of reducing. Especially when PEG-s-PEI degraded fast in pH 5.0, there was no time for IND to reassemble with retained PEI chains, leading the drug release from NAs highly accelerated. Related to the different degradation speeds of PEG-s-PEI under various pH conditions (Fig. S2), DTX/IND/PEG-s-PEI NAs exhibited an excellent pH-triggered release behavior at pH 5.0 stimulating pH in lysosomes, while it was slightly faster DTX release at pH 6.5 stimulating pH_e_ in tumor microenvironment than pH 7.4 (Fig. 5a). Apparently, DTX/IND/PEG-s-PEI NAs exhibited more excellent pH-triggered release and PEGylation cleavage behaviors at pH 5.0, and only pH-triggered PEGylation cleavage at pH 6.5 with little change at drug release behavior than pH 7.0. These behaviors provided the basis for NAs to firstly PEGylation deshielded in tumor microenvironment and then be delivered into tumor cells. Benefit of PEGylation, DTX/IND/PEG-s-PEI NAs and DTX/IND/PEG-b-PEI NAs showed obvious long circulation behaviors at 10 mg/kg of DTX than raw DTX *in vivo* (Fig. 5b), which resulted in remarkably enhanced t_1/2_ (Fig. 5c).

Studies on *in vivo* antitumor activities of various formulations were conducted that mice received DTX/IND/PEG-s-PEI NAs showed significantly enhanced anti-tumor activity compared with raw DTX and DTX/IND/PEG-b-PEI NAs (Fig. 6a–c). Meanwhile, the anorexia induced by docetaxel-based chemotherapy was controlled that body weights of mice received IND-DTX combined nanotherapies were higher than mice received raw DTX (Fig. 6d). These results demonstrated the superior efficacy of newly assembled carriers for targeted delivery of hydrophobic drugs for tumor therapy based on tumor microenvironment. Importantly, this system performed a good safety that no significant toxicity of DTX/IND/PEG-s-PEI NAs was observed in 14 days treatment (Figs 6d–f and S6a–c), and PEG-s-PEI seems to be safe in cytotoxicity even at dose of 20 µg/mL (Fig. S2c,d) both in pH 6.5 and pH 7.4 mediums, which is much higher than that to be used in *in vivo* evaluations and therapy. However, comprehensive and long term toxicity evaluation is necessary to further study the potential toxicity of accumulated free PEI derived from PEG-s-PEI *in vivo*.

In summary, a pH-sensitive PEGylation cleavable PEGylated PEI (PEG-s-PEI) was successfully synthesized, and DTX was highly efficiently packaged into NAs driven by multiple noncovalent interactions-mediated host-guest assembly of IND and PEG-s-PEI. These NAs, with desirable redispersibility and scalability, exhibited excellent pH-sensitivity PEGylation cleavage performance between pH_e_ of tumor microenvironment and normal tissues. By being PEGylation selectively cleaved under the acidy conditions in tumor microenvironment, these NAs exhibited a significantly charge shift and highly efficient phagocytosis by tumor cells, while long blood circulation time was retained by PEG chains stretching in circulation. This smart pH-sensitive PEGylation cleavage provided an efficient strategy to target tumor microenvironment, in turn afforded superior therapeutic outcome in anti-tumor activity. Compared with other strategy to target tumor site, this delivery strategy performed several advantages containing prolonged circulation, accumulation in tumors, highly efficient cellular internalization and rapid intracellular drug release. More importantly, this delivery system could be facile fabricated by one-pot assembly and universal drugs applicable, which is providing a new strategy to fabricate the next generation of drug delivery systems and will achieve better therapeutic effects in cancer treatment.

## Methods

### Materials

Methoxyl polyethylene glycol (mPEG) with Mw of 2 kDa, mPEG-NHS (MW = 2000), branched polyethyleneimine (PEI) with Mw of 25 kDa (PEI), indomethacin (IND) were obtained from Sigma (USA). 4-Formylbenzoic was purchased from Aesar Alfar (Ward Hill, MA, USA). Cremophor EL was purchased from BASF Corp (Germany). Docetaxel (DTX) were supplied by Xi’an Xuan Biological Technology Co., Ltd (Xi’an, China). Penicillin, streptomycin, fetal bovine serum (FBS), and Eagle’s minimum essential (EME) medium were purchased from HyClone (Waltham, MA, USA). RPMI1640 medium was obtained from Gibco (USA). All the other reagents are commercially available and used as received.

### Synthesis of PEGylated PEIs

The synthesis of PEGylated PEI linked by Schiff base (PEG-s-PEI) was achieved via a two-step process (Fig. S1a). mPEG-CHO was firstly synthesized by mPEG (MW = 2000) and 4-Formylbenzoic. Then the reaction was carried at various PEG/PEI monomeric molar ratios for PEG-s-PEI-1 (2/1), PEG-s-PEI-2 (4/1),and PEG-s-PEI-3 (8/1). After reaction, the solution was concentrated, the resulting mixture was transferred to a dialysis membrane (MWCO: 3,500) against distilled water for 2 days. And PEG-s-PEIs were freeze dried from dialyzed solution. PEGylated PEI linked by amide linkage (PEG-b-PEI) was synthesized as previous report by mPEG-NHS (MW = 2000) and PEI at monomeric molar ratio of 4/1^49^. The number of PEG linked at PEI was calculated from ^1^H NMR spectra by the integral intensities of the signals at 3.38 ppm (CH_3_O-) and signals at 2.3–3.3 ppm (-CH_2_CH_2_-NH-). PEG-s-PEIs were firstly solved in water, and separated on the cation-exchange column, the amounts of unreacted PEG were pooled. Then, the amount of PEG was quantified by measuring the absorbance at 340 nm, the main absorbance of the released aldehyde.

### Hydrolysis Assay

The PEG-s-PEIs were adjusted to pH 5.5, 6.0, 6.5 and 7.4 by the addition of acetic acid to a final acetate concentration of 0.2 M total volume 2 mL and was incubated for 5 h at 37 °C. The reaction was applied to and separated on the cation-exchange column, the unbound PEG fractions were pooled, and the amount of PEG was quantified at different time points by measuring the absorbance at 340 nm.

### Computational Studies

Polymers with 50 units were built in three-dimensional (3D) coordinates using MOE’s (Molecular Operating Environment software package, Chemical Computing Group, Canada) builder tool. And 5 units was employed to build as small version of polymer to stimulate self-inter-molecular interaction in docking process. The 3D structures of IND, polymer chains, small version of polymer and water molecular were preoptimized before running simulations using the all atom MMFF94x force field with no constraints. For all docking calculations, the size of the grids was set at 126 × 126 × 126 Å using grid spaces at 0.375 was probed to find the most favorable drug-polymer complex geometry (interactions), Lamarckian Genetic Algorithm (LGA) built in Autodock4.2 was probed with 100 docking runs, and LGA searching algorithms the number of energy evaluation was set to 25,000,000 while the population size was set to 150. The other docking parameters were set to the default values. The docking results were analysed by Autodock tools (ADT 1.56) and MOE, while interactions bewteen polymer/repeat unit was estimated by small version polymer docked to polymer and caluated by the unit with highest interaction energy.

### Fabrication of Nanoassemblies

NAs were prepared by a dialysis procedure. Briefly, DTX, IND and polymer (PEG-s-PEIs, PEG-b-PEI or PEI) at different feeding ratios were dissolved in methanol. The polymer concentration was 10 mg/mL. Thus obtained solution was dialyzed against deionized water at 25 °C. The outer aqueous solution was exchanged every 2 h. After 24 h of dialysis, DTX/IND/polymer NAs were attained and collected for analysis without further treatment. The drug content in the lyophilized samples was quantified by high performance liquid chromatography (HPLC). The drug loading content and entrapment efficiency were calculated according to the following equations:

1 $${\rm{Loading}}\,{\rm{content}}\,( \% )=\frac{{\rm{The}}\,{\rm{weight}}\,{\rm{of}}\,{\rm{drug}}\,{\rm{in}}\,{\rm{nanoassemblies}}\,(\mathrm{mg})}{{\rm{The}}\,{\rm{weight}}\,{\rm{of}}\,{\rm{nanoassemblies}}\,(\mathrm{mg})}\times \mathrm{100} \% $$

### *In vitro* Hemolysis Assay

Various NAs were mixed with whole blood and incubated for 45 minutes at 37 °C. The cells are centrifuged and the absorbance of the supernatant, which includes plasma and lysed erythrocytes, is measured. Percent lysis is calculated from a standard curve of lysed erythrocytes.

### *In vitro* pH triggered potential shift and particle size changes

Particle size and ζ-potential of NAs incubated in various buffers were measured at determined time point. Measurements at pH 7.4 were performed in PBS (0.01 M, pH 7.4), while for pH 5.5 and pH 6.5 measurements, pH values were adjusted by the addition of acetic acid to a final acetate concentration of 0.2 M total volume 2 mL.

### Intracellular uptake of NAs

B16F10 and HepG2 cells were separately seeded in a 24-well plate with a density of 1.0 × 10^5^ cells per well in 500 μL growth medium at pH 6.5 or 7.4 adjusted by the addition of acetic acid. Cells were incubated at 37 °C with 5% CO_2_ for 24 h. Then the culture medium was replaced by 500 μL of fresh medium (pH 6.5 or pH 7.4) containing DTX/IND/PEG-s-PEI NAs (fabricated based on a formulation with DTX/IND/PEG-s-PEI weight ratio of 10:10:10), respectively. After incubation for various times, cells were washed with PBS and lysed. The drug content was measured by HPLC, while the content of total proteins was quantified by a BCA method. The content of cellular drug was normalized to the protein content.

### *In vitro* release study

For *in vitro* release tests, 0.5 mL of freshly prepared NAs was placed into dialysis tubing (MWCO: 3500 Da), which was immerged into 40 mL of PBS (0.01 M) at pH 5.5, 6.5 or 7.4. At predetermined time intervals, 4.0 mL of release medium was withdrawn, and the same volume of fresh PBS was supplemented. DTX concentration in the release buffer was quantified by HPLC.

### *In vivo* pharmacokinetic study

All the animal care and experimental protocols were approved by the local animal ethics committee at Third Military Medical University, and performed in compliance with the Animal Management Rules of the Ministry of Health of the People’s Republic of China (No. 55, 2001) and the guidelines for the Care and Use of Laboratory Animals of Third Military Medical University. Fifteen male Sprague-Dawley (SD) rats (200–250 g) were randomly assigned into three groups of five animals each. DTX/IND/PEG-s-PEI NAs, DTX/IND/PEG-b-PEI NAs and pristine DTX (solubilized in the aqueous solution containing Cremophor EL and ethanol) were intravenous injected (*i.v*.) at a dose of 10.0 mg/kg, respectively. All the rats fasted overnight before administration. Blood samples were collected at specific time points post-dose. DTX concentration in plasma was quantified by HPLC. The t_1/2_ time was calculated with non-compartment method by DAS 3.0.

### *In vitro* antitumor evaluation

B16F10, HepG2, MCF-7, and MDA-MB-231 cells were cultured in 96-well plates with a density of 1.0 × 10^4^ per well in 100 μL growth medium containing 10% (v/v) FBS, 100 U/mL of penicillin and 100 μg/mL of streptomycin. All cells were incubated at 37 °C in a humidified atmosphere of 5% CO_2_ for 24 h before the addition of DTX-containing NAs. Cells were then treated with the medium at various pH values (5.5, 6.5 or 7.4) containing DTX/IND/PEG-s-PEI, DTX/IND/PEG-b-PEI NAs, or pristine DTX (solubilized in the aqueous solution containing Cremophor EL and ethanol) at different doses for 24 h. The cell viability was quantified by MTT method. B16F10, HepG2, and MDA-MB-231 cells were cultured in RPMI 1640 medium, while MCF-7 cells were cultured in EME medium.

### Assessment of antitumor activity of DTX-containing assemblies

Tumor xenografts were generated by inoculating B16F10 murine melanoma cells into the right limb armpits of athymic nude mice. After 10 days, when the average volume of the xenograft tumors reached 50 mm^3^, the mice were randomly divided into 4 groups (n = 6), for which saline and various nanoassembiles formulations of DTX/IND/PEG-s-PEI,DTX/IND/PEG-b-PEI, and pristine DTX were administered (*i.v*) every four days at the DTX dose of 10 mg/kg, respectively. After 15 days, all mice were sacrificed, and the collected tumors were weighed, washed with cold saline, dried on filter paper, weighed and cut into small pieces. The amount of DTX in the tumor was determined by HPLC as previously reported. Blood samples and main organs were also collected for further analysis.

### Measurements

^1^H NMR spectra were recorded on a Varian INOVA-400 spectrometer operating at 400 MHz. FT-IR spectra were acquired on a Perkin-Elmer FT-IR spectrometer (100 S). Gel permeation chromatography (GPC) measurement was carried out using a Waters model 440, equipped with a Wyatt Optilab Refractive Index detector. Dynamic light scattering (DLS) and ζ-potential measurements were performed on a Malvern Zetasizer Nano ZS instrument. The freshly prepared samples were diluted according to their scattering intensities for size determination. Unless stated otherwise, measurements were implemented at 25 °C. Transmission electron microscopy (TEM) observation was carried out on a TECNAI-10 microscope (Philips, Netherland) operating at an acceleration voltage of 80 kV.

## Electronic supplementary material

Supplementary material: Smart pH-sensitive nanoassemblies with cleavable PEGylation for tumor targeted drug delivery
