# Supplementary material for: Smart pH-sensitive nanoassemblies with cleavable PEGylation for tumor targeted drug delivery
Source: Sci Rep. 2017 Jun 13;7:3383. doi: 10.1038/s41598-017-03111-2 (PMC5469818; doi:10.1038/s41598-017-03111-2)
Supplement: Supplementary file 1 — Supplementary material: Smart pH-sensitive nanoassemblies with cleavable PEGylation for tumor targeted drug delivery [file 41598_2017_3111_MOESM1_ESM.doc]

**Supplementary material:**

Smart pH-sensitive nanoassemblies with cleavable PEGylation for tumor targeted drug delivery

Guanren Zhao^a,‡^, Ling Long^b,‡^,Lina Zhang^a^, Mingli Peng^a^, Ting Cui^a^, Xiaoxun Wen^a^, Xing Zhou ^c,*^, Lijun Sun^a^,*, Ling Che^a^, *

^a^Department of Pharmacy, Hospital 309 of PLA, Beijing 100091, China

^b^Department of oncology, Xinqiao Hospital, Third Military Medical University, Chongqing 400038, China

^c^Department of Pharmaceutics, College of Pharmacy, Third Military Medical University, Chongqing 400038, China.

^‡^These authors contributed equally to this work.

* Corresponding authors:

Ling Che, PhD

Email: cheling309@126.com

Lijun Sun, Prof

Email: joycesun1973@126.com

Xing Zhou, PhD

Email: diszhou@126.com

**Table S1.** Molecular weight and molecular weight distribution of various polymers

| **Polymers** | **Mw** | **Mn** | **Mw/Mn** |
| --- | --- | --- | --- |
| PEI | 26000 | 19000 | 1.39 |
| PEG-b-PEI | 91000 | 74000 | 1.23 |
| PEG-s-PEI-1 | 78000 | 60000 | 1.30 |
| PEG-s-PEI-2 | 88000 | 69000 | 1.27 |

**Table S2.** Hemolysis Ratio of NAs (data presented as mean ± SD, n = 3)

| **NAs** | **Hemolysis ratio(%)** |
| --- | --- |
| DTX/IND/PEI | 81.234±1.343 |
| DTX/IND/PEG-b-PEI | 1.561±0.351 |
| DTX/IND/PEG-s-PEI-1 | 11.335±1.564 |
| DTX/IND/PEG-s-PEI-2 | 3.231±0.822 |


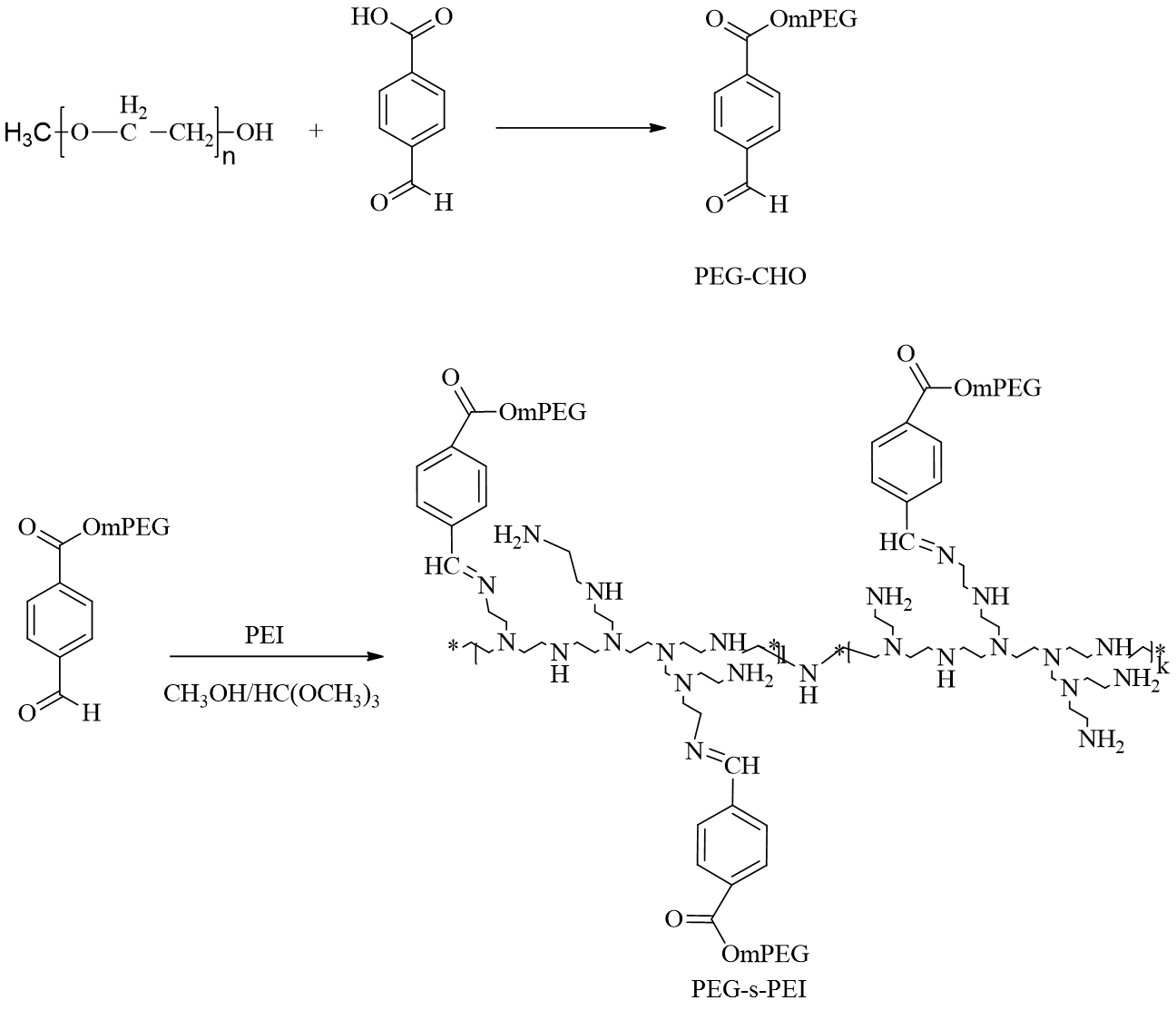


a


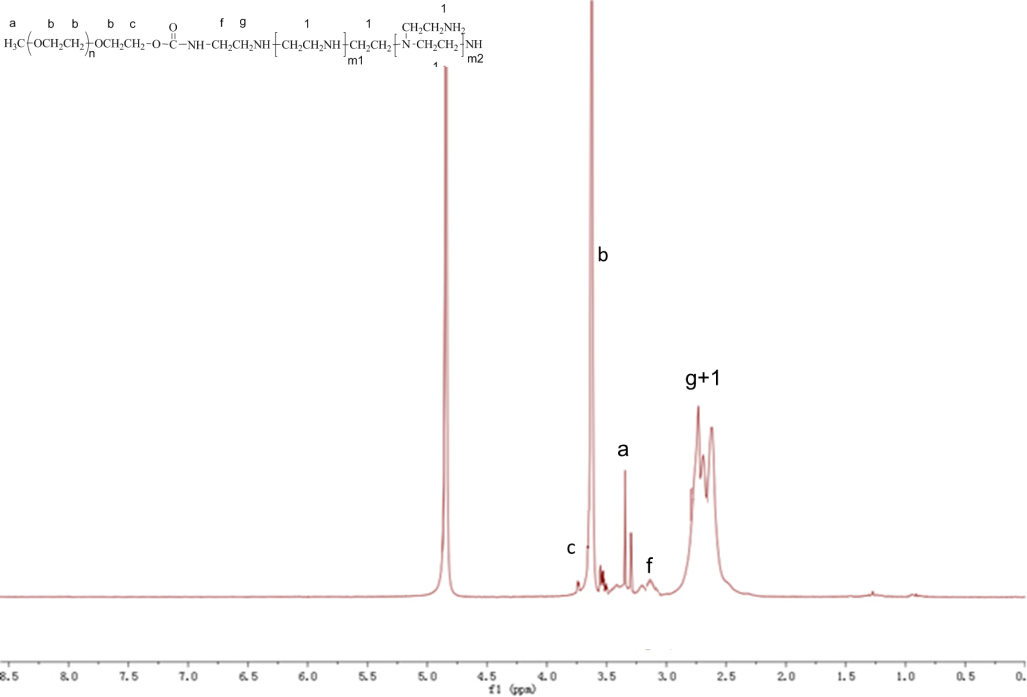


b c


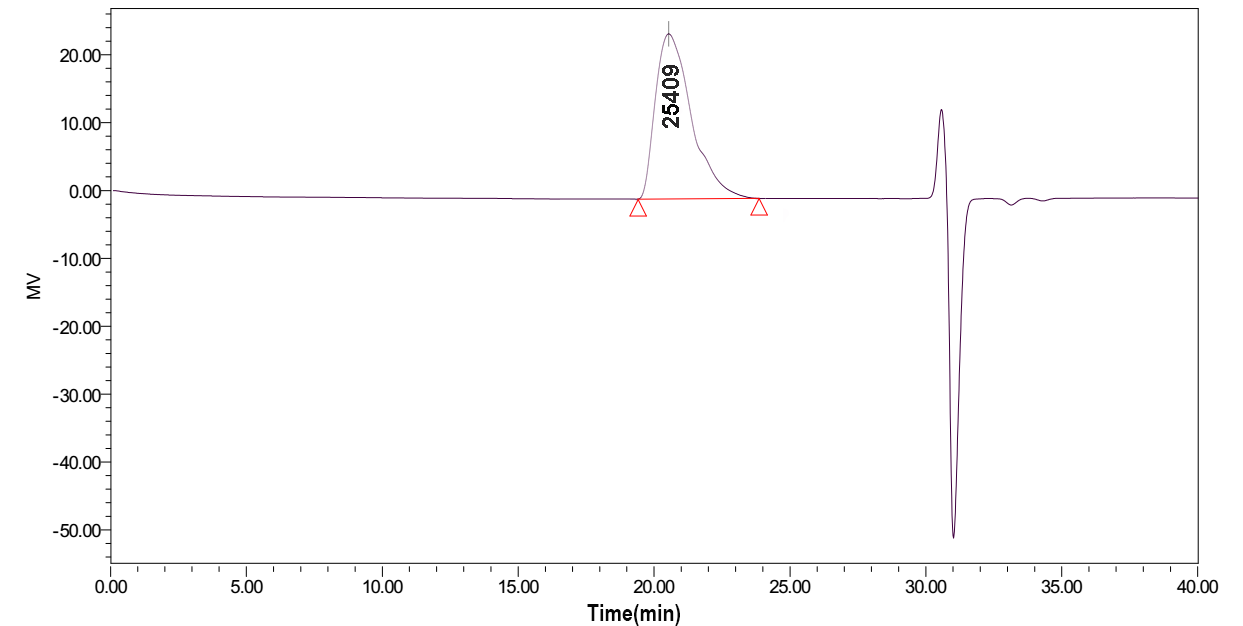


d


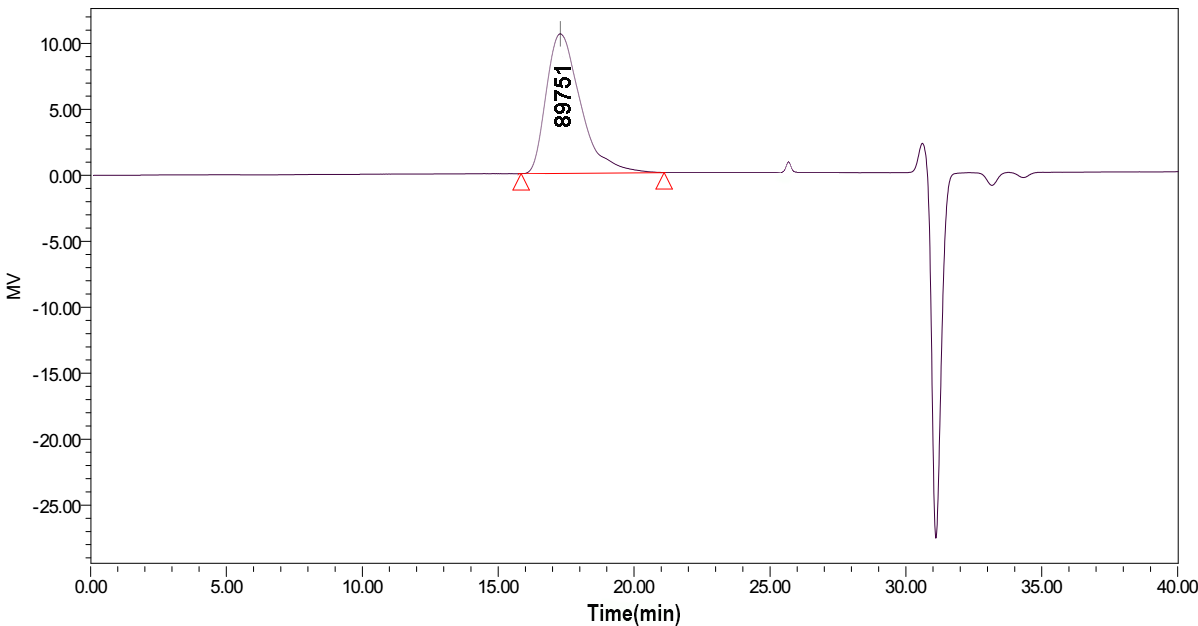


e


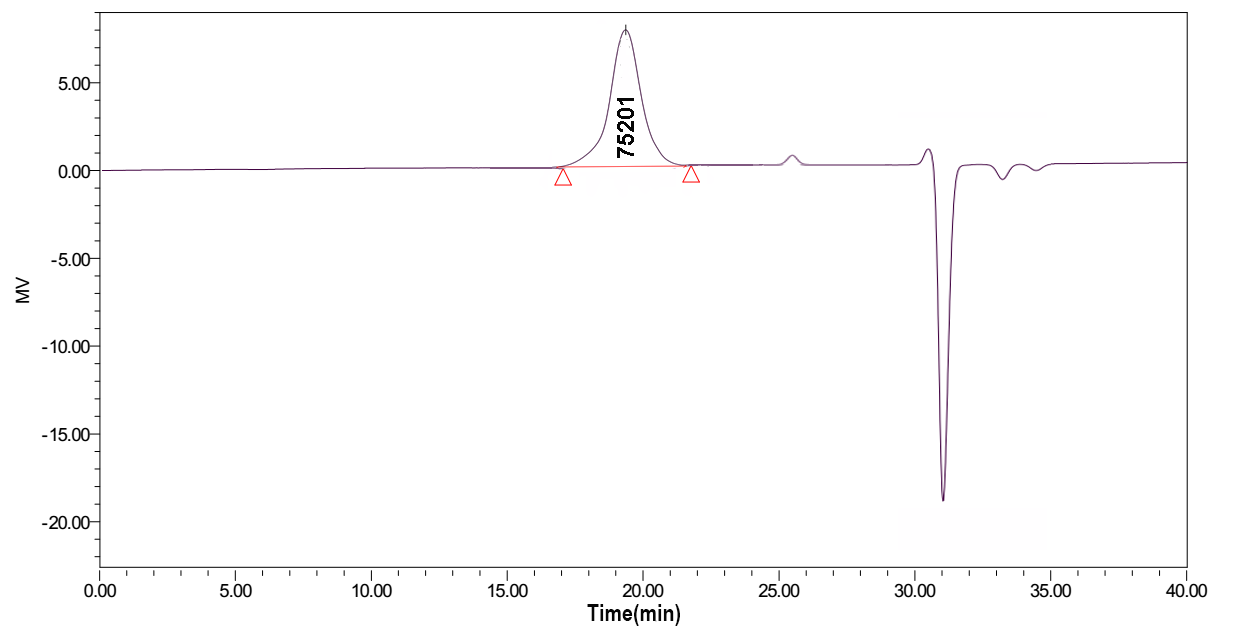


f


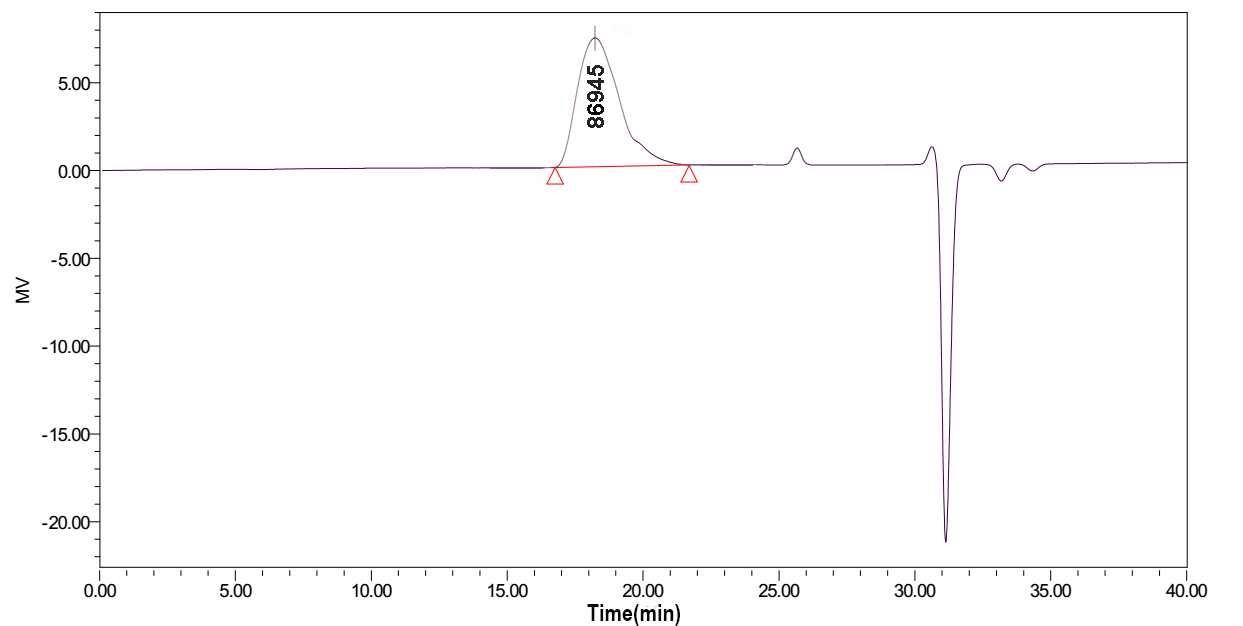


g

Figure S1. **a,** Synthetic route for preparation of PEG-s-PEI conjugates. **b-c,** Representative ^1^H-NMR spectrum (600 MHz) of PEG-CHO(a) and PEG-b-PEI (b).**d-f,** GPC spectrum of PEI(d),PEG-b-PEI(e), PEG-s-PEI-1(f) and PEG-s-PEI-2(g).

**Note:** mPEG-CHO was firstly synthesized by mPEG(0.194 mmol) and 4-Formylbenzoic(0.23mmol) in DCM (5 mL). Then, DMAP(0.053 mmol), EDCI (0.21 mmol) and Et_3_N (0.21 mmol) were added and stirred for 24 h at room temperature, and then transferred to a dialysis membrane (MWCO: 1,000) against distilled water for 2 days. The dialyzed solution was freeze-dried to obtain mPEG-CHO. Then the reaction was carried at various PEG/PEI monomeric molar ratios for PEG-s-PEI-1 (2/1) and PEG-s-PEI-2(4/1) that PEI(0.0097 mmol) or PEI (0.0049 mmol) was added to a solution of PEG-CHO(0.194 mmol) in 5mL of methanol/ Triethyl orthoformate (10/1), and stirred for 48h at room temperature. After reaction, the solution was concentrated, the resulting mixture was transferred to a dialysis membrane (MWCO: 3,500) against distilled water for 2 days. PEG-s-PEIs were freeze dried from dialyzed solution.

a b

c d

**Figure S2. a-b,**Time course of the cleavage of PEG from PEG-s-PEI-1(a) and PEG-s-PEI-2(b). c-d, Cytotoxicity evaluations of PEI and PEG-s-PEI-2 against HepG2 in pH 7.4 medium(c) and pH 6.4 medium(d). After 6 h of incubation with various concentrations of related materials, cells were incubated for additional 24 h, and then cell viability was determined by MTT assay. Data are mean ± S.E. (n = 6).

**Figure S3.** IND loading contents in nanoassemblies

a b

c d

e f

g h

**Figure S4.** Cellular retention of internalized drug in tumor cells after endocytosis. **a-b,** Time dependent DTX concentration changes after raw DTX endocyozied by B16F10(a) and HepG2(b) in normal and acidy mediums. Data are mean ± SD (n = 3). **c-d,** Time dependent DTX concentration changes after DTX/IND/PEG-b-PEI nanoassemblies endocyozied by B16F10(c) and HepG2(d) in normal and acidy mediums. **e-f,** Time dependent DTX concentration changes after DTX/IND/PEI nanoassemblies endocyozied by B16F10(e) and HepG2(f) in normal and acidy mediums. **g-h,** DTX concentration in B16F10(g) and HepG2(h) cells 12 hours after endocytosis of various nanotherapeutics. Data are mean ± SD (n = 3),** means pH 7.4 vs pH 6.5 , p<0.01 .

.

a

b

c d

e

**Figure S5.** *In vitro* antitumor activity of DTX nanomedicines formulated from DTX/IND/PEG-s-PEI assemblies against various cancer cells in normal and acidy mediums. **a,** Resistant MCF-7 human breast cancer cells. **b,** Resistant MDA-MB-231 human breast cancer cells. **c-d,** The IC50 values of DTX formulations against various tumor cells in pH 7.4 medium(c) and pH 6.5 medium(d) after 12 h. **e,** The IC50 values of DTX/IND/PEG-s-PEI against various tumor cells in pH 7.4 medium and pH 6.5 medium after 12 h.Data are mean ± S.E. (standard error, n = 6). DTX/IND/PEG-b-PEI and DTX/IND/PEG-s-PEI nanomedicines at weight ratio of 10:10:10 was employed in these cell culture experiments. It should be noted that the effect of IND/PEG-s-PEI vehicle was eliminated by using corresponding controls in all experiments. *, p < 0.05, **, p < 0.01, ***, p < 0.001 compared with the saline control.

**
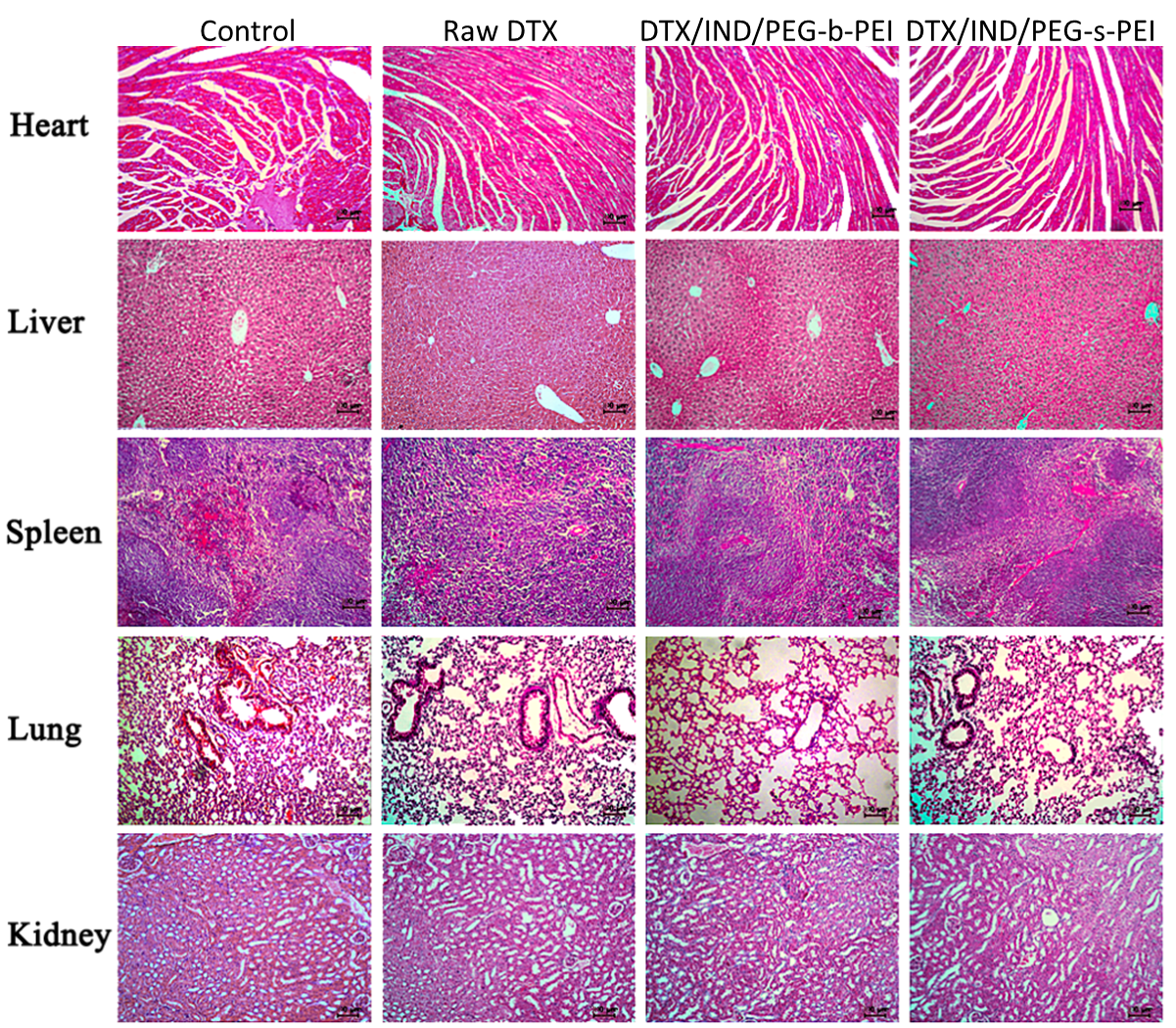
**

a

b

c

**Figure S6.** **a,** H&E sections of main organs from mice after various treatments. **b.** The organ index of mice after 14 days of DTX treatment. **c,** Hematological parameters of blood samples from mice subjected to various treatments( WBC, HGB, RBC and PLT). Results are expressed as mean ± S.D. (n = 12 in body weight, while 6 in others).
